# Comparative efficacies of various corticosteroids for preventing postextubation stridor and reintubation: a systematic review and network meta-analysis

**DOI:** 10.3389/fmed.2023.1135570

**Published:** 2023-07-24

**Authors:** I-Jung Feng, Jia-Wei Lin, Chih-Cheng Lai, Kuo-Chen Cheng, Chin-Ming Chen, Chien-Ming Chao, Ying-Ting Wang, Shyh-Ren Chiang, Kuang-Ming Liao

**Affiliations:** ^1^Institute of Precision Medicine, National Sun Yat-sen University, Kaohsiung, Taiwan; ^2^Department of Medical Research, Chi Mei Medical Center, Tainan, Taiwan; ^3^Division of Hospital Medicine, Department of Internal Medicine, Chi Mei Medical Center, Tainan, Taiwan; ^4^Department of Internal Medicine, Chi Mei Medical Center, Tainan, Taiwan; ^5^Department of Safety, Health, and Environmental Engineering, Chung Hwa University of Medical Technology, Tainan, Taiwan; ^6^Departments of Intensive Care Medicine, Chi Mei Medical Center, Tainan, Taiwan; ^7^Recreation and Health-Care Management, Chia Nan University of Pharmacy and Science, Tainan, Taiwan; ^8^Department of Intensive Care Medicine, Chi Mei Medical Center, Liouying, Taiwan; ^9^Departments of Respiratory Therapy, Chi Mei Medical Center, Tainan, Taiwan; ^10^Department of General Education, Chia Nan University of Pharmacy and Science, Tainan, Taiwan; ^11^Department of Internal Medicine, Chi Mei Medical Center, Chiali, Taiwan

**Keywords:** dexamethasone, hydrocortisone, methylprednisolone, network meta-analysis, postextubation stridor, reintubation

## Abstract

**Objectives:**

We assessed the efficacies of various corticosteroid treatments for preventing postexubation stridor and reintubation in mechanically ventilated adults with planned extubation.

**Methods:**

We searched the Pubmed, Embase, the Cochrane databases and ClinicalTrial.gov registration for articles published through September 29, 2022. Only randomized controlled trials (RCTs) that compared the clinical efficacies of systemic corticosteroids and other therapeutics for preventing postextubation stridor and reintubation were included. The primary outcome was postextubation stridor and the secondary outcome was reintubation.

**Results:**

The 11 assessed RCTs reported 4 nodes: methylprednisolone, dexamethasone, hydrocortisone, and placebo, which yielded 3 possible pairs for comparing the risks of post extubation stridor and 3 possible pairs for comparing the risks of reintubation. The risk of postextubation stridor was significantly lower in dexamethasone- and methylprednisolone-treated patients than in placebo-treated patients (dexamethasone: OR = 0.39; 95% CI = 0.22–0.70; methylprednisolone: OR = 0.22; 95% CI = 0.11–0.41). The risk of postextubation stridor was significantly lower in methylprednisolone-treated patients than in hydrocortisone-treated: OR = 0.24; 95% CI = 0.08–0.67) and dexamethasone-treated patients: OR = 0.55; 95% CI = 0.24–1.26). The risk of reintubation was significantly lower in dexamethasone- and methylprednisolone-treated patients than in placebo-treated patients: (dexamethasone: OR = 0.34; 95% CI = 0.13–0.85; methylprednisolone: OR = 0.42; 95% CI = 0.25–0.70). Cluster analysis showed that dexamethasone- and methylprednisolone-treated patients had the lowest risks of stridor and reintubation. Subgroup analyses of patients with positive cuff-leak tests showed similar results.

**Conclusions:**

Methylprednisolone and dexamethasone were the most effective agents against postextubation stridor and reintubation.

## Introduction

Endotracheal intubation is a common procedure for critically ill patients with acute respiratory failure that requires mechanical ventilation (MV) in an intensive care unit (ICU) or for patients under general anesthesia undergoing surgery in the operating room. Although endotracheal intubation is usually uneventful, it can lead to tracheal rupture, pneumothorax or pneumomediastinum, tongue necrosis, or soft palate injury (1–5). Laryngeal edema is another complication of endotracheal intubation, and it might be a cause of postextubation stridor and subsequent reintubation (6). Most important, laryngeal edema-associated extubation failure can prolong an ICU or hospital stay, increase mortality and morbidity, and add to medical costs. Thus, it is important to avoid laryngeal edema-associated postextubation stridor and reintubation failure.

Several interventions—parenterally administering corticosteroids, nebulizing epinephrine, and administering an inhaled helium/oxygen mixture—have been developed to prevent postextubation stridor and reintubation (6). Corticosteroids are supposed to reduce both inflammation and edema, and to exert their effect on preventing postextubation stridor and reintubation. Moreover, several meta-analyses (7–9) have shown that administering prophylactic corticosteroids before planned extubation significantly reduced the incidence rates of postextubation stridor and reintubation in adults. In studies that used methylprednisolone, dexamethasone, hydrocortisone, and placebo (10–19), however, corticosteroid anti-inflammation potencies differed. No prior study has specifically compared these four interventions. We hypothesized that different corticosteroids would have different effects against postexubation stridor and reintubation. Therefore, we did this network meta-analysis to assess the efficacies of these interventions.

## Methods

### Study search and selection

This network meta-analysis (NMA) was based on the Preferred Reporting Items for Systematic Reviews and Meta-analyses (PRISMA) extension statement (20). The protocol of this study was prospectively registered at PROSPERO (registration number: CRD42022362888). All clinical studies were identified in a systematic review of the literature through September 29, 2022 in the Pubmed, Embase, the Cochrane databases and ClinicalTrial.gov registration. We used the following search terms: “intubation,” “intratracheal,” “laryngeal edema,” “airway obstruction,” “stridor,” “post extubation larynx^*^ edema^*^,” steroid^*^,” “corticosteroid^*^,” ‘glucocorticoid^*^,” “prednisone,” “prednisolone,” “methylprednisolone,” “dexamethasone,” “cortisone,” “hydrocortisone,” and “adult” (Appendix 1). Only randomized clinical trials (RCTs) that compared the efficacy of systemic corticosteroid treatments and other interventions against postextubation stridor and reintubation in MV adults scheduled for extubation were included. We also searched for additional eligible studies in the reference lists of retrieved articles and relevant meta-analyses. To avoid bias, two reviewers (CCL and JWL) independently searched and examined publications. Any discrepancies were judged and determined by third author (IJF).

### Data extraction

The following data were extracted from every included study: year of publication, study design, sample size, gender ratio, treatment regimens of corticosteroids and other interventions, cumulative doses (mg), frequency of administration, and incidence rates of postextubation stridor and reintubation. For RCTs that used medians and interquartile ranges as measures of central tendency and dispersion, we estimated means and standard deviations (SDs) (21).

### Definitions and outcomes

The primary outcome was overall postextubation stridor and reintubation. Subgroup analyses of patients with positive cuff-leak tests were also performed.

### Quality assessments

The quality of the analyzed RCTs and the risk of bias were assessed using the Cochrane Risk of Bias Assessment tool (22). We assessed seven quality domains: sequence generation, allocation concealment, participant masking, personnel masking, outcome assessor masking, incomplete outcome data, and reporting bias. Other biases were also assessed. Finally, we judged the quality of each domain as (a) low risk of bias, (b) unclear risk of bias, or (c) high risk of bias.

### Statistical methods

We used STATA 15.0 (StataCorp, College Station, TX, USA), R (version 4.2.1) and R package “netmeta” to do a frequentist network meta-analysis (23). Because of the diversity of the task-related characteristics of the included RCTs, we used a random-effects model. The heterogeneity variable of all comparisons was assumed to be a single τ^2^ value. The required property of transitivity was also assumed.

We used a design-by-treatment interaction model for global tests and side-splitting model for local tests to evaluate inconsistencies between direct and indirect evidence. Network plots show direct comparisons of investigated outcomes, and odds ratios (ORs) and 95% confidence intervals (95% CIs) show network effect-size estimates of pairwise comparisons.

A surface under the cumulative ranking curve (SUCRA) presents the likely rank order of each investigated treatment. To evaluate the joint ranking of postextubation stridor and reintubation both clinical outcomes, cluster analysis was performed. The optimal number of resulting cluster was defined by having maximum intra-cluster similarity and minimize inter-cluster similarity (24). Finally, we assessed publication bias using Egger's test.

## Results

### Search results and characteristics of the included studies

**Figure 1:**
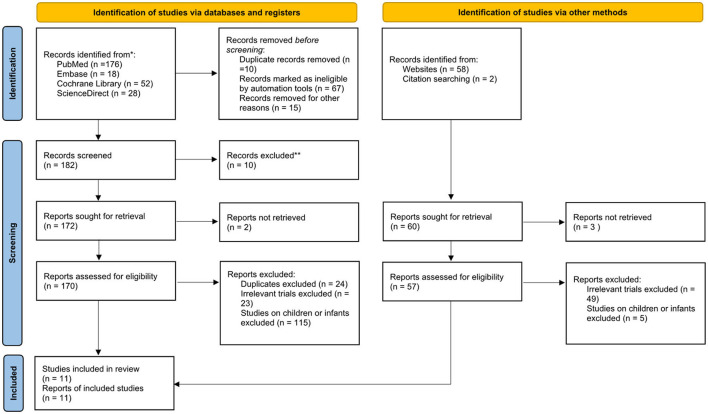
The algorithm of study selection.

We initially identified 308 studies. After excluding 44 duplicate articles, 264 articles were screened, 135 of which were excluded on the basis of the title and abstract. After excluding study on non-adult patients (*n* = 135), non-RCT (*n* = 30), the 99 remaining articles underwent a full-text review to assess their eligibility. Finally, a total of eleven RCTs (10–19, 25) fulfilled the inclusion criteria were selected for our network meta-analysis (Figure 1; Table 1). Six RCTs (11, 12, 16–19) were done in Taiwan, three (13–15) in France, one (25) in India, and one (10) in Pakistan. Six RCTs (10–12, 17–19) used the cuff leak test (CLT) to select high-risk patients. Dexamethasone was the most commonly used corticosteroid (*n* = 5), (10, 13, 17, 18, 25) then methylprednisolone (*n* = 4), (11, 12, 14, 15) and, finally, hydrocortisone (*n* = 2) (16, 19). Follow-up observation durations were 24 h (13, 14, 16, 25) and 48 h (10–12, 17, 18). Routes of administration, doses, and frequencies of administration differed (Appendix 2). Four studies (11, 13, 15, 16) used one injection before extubation, but five studies (10, 12, 17–19) used corticosteroid for 24 h before extubation. One RTC (25) intravenously injected one group of patients with dexamethasone and treated another group with nebulized budesonide 1 h before the scheduled extubation, and then every 12 h for 48 h after the extubation.

**Table 1:** Characteristics of included studies.

**Study/year**	**Location**	**Inclusion criteria**	**Study duration**	**Pretest with Cuff leak test**	**Sample Size (% of female)**	**Age, mean ±SD**	**Corticosteroid Regimen**	**Comparator**	**Observation period after extubation (h)**
Gaussorgues et al. (15)	France	1. Tracheal intubation	1985/9–1986/8	–	276 (65.22)	54.00 ± 24.55	Methylprednisolone	NR	NR
Darmon et al. (13)	France	1. Tracheal intubation	1986/11–1987/11	–	700 (42.14)	53.15 ± 19.54	Dexamethasone	placebo	24
Ho et al. (16)	Taiwan	1. Intubation≥24 h and 2. Met the weaning criteria	1990/3/1–1990/8/31	–	77 (23.38)	62.48 ± 16.10	Hydrocortisone	N/S	24
Cheng et al. (12)	Taiwan	1. ≥18 yrs of age 2. intubation≥24 hrs 3. CLV% < 24% 4. Met the weaning criteria	2002/2–2004/7	+	127 (62.20)	66.15 ± 16.58	Methylprednisolone	N/S	48
Francois et al. (14)	France	1. ≥18 yrs of age 2. MV≥36 h	2001/3– 2002/1	–	761 (36.40)	62.25 ± 20.69	Methylprednisolone	Isotonic saline	24
Lee et al. (17)	Taiwan	1. ≥18 years of age. 2. MV >48 h 3. CLV ≤ 110 ml 4. Met the weaning criteria	2004/10–2006/3	+	80 (82.50)	72.55 ± 14.26	Dexamethasone	Placebo	48
Shih et al. (26)	Taiwan	1. MV ≥ 24 h 2. Met the weaning criteria 3. CLV < 110 ml	NR	+	98 (44.90)	NR	Hydrocortisone	N/S	NR
Malhotra et al. (25)	India	1. MV≥24 h 2. Met the weaning criteria and first extubation	2003/1–2006/2	–	60 (46.67)	32.90 ± 14.14	Dexamethasone	Placebo or saline	24
Baloch et al. (10)	Pakistan	1. ≥18 years of age. 2. MV≥48 h 3. CLV ≤ 110 ml	2006/8–2008/7	+	92 (44.57)	39.65 ± 12.65	Dexamethasone	N/S	48
Cheng et al. (11)	Taiwan	1. ≥18 yrs of age 2. intubation≥4 h 3. Met the weaning criteria 4. CLT < 24%	NR	+	71 (77.46)	60.49± 16.74	Methylprednisolone	N/S	48
Lin et al. (27)	Taiwan	1. ≥18 yrs of age 2. MV≥48hrs 3. CLV < 110ml 4. Met the weaning criteria	2007/4/1–2010/3/31	+	126 (78.57)	74.09 ± 11.80	Dexamethasone	N/S	48

NR, not reported; CLT, cuff leak test; MV, mechanical ventilation; CLV, cuff leak volume; N/S, normal saline.

### Network meta-analysis

**Figure 2:**
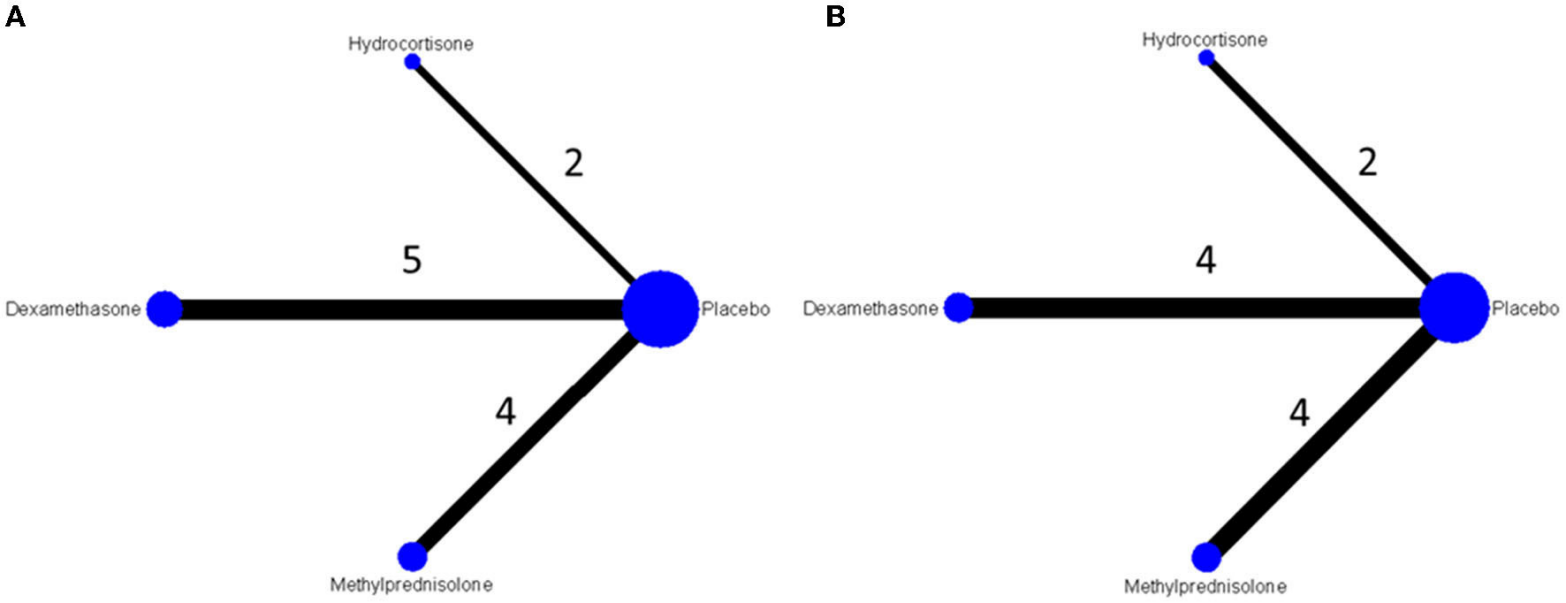
Network plot for preventive effect of **(A)** postextubation stridor and **(B)** reintubation.

Among enrolled studies, four reported nodes were methylprednisolone, dexamethasone, hydrocortisone and placebo, resulting in 3 possible pairs for comparing the risks of post extubation stridor and 3 possible pairs for comparing the risks of reintubation. Most comparisons with direct evidence are from dexamethasone or methylprednisolone and placebo. Regarding the risk of postextubation stridor, five studies compared the effects between dexamethasone and placebo. Four and one studies used methylprednisolone and hydrocortisone as study agents, respectively (Figure 2A). Regarding the risk of reintubation, each four studies compared the effect between dexamethasone or methylprednisolone and placebo. Two studies used compared hydrocortisone with placebo (Figure 2B).

### Quality of studies

**Figure 3:**
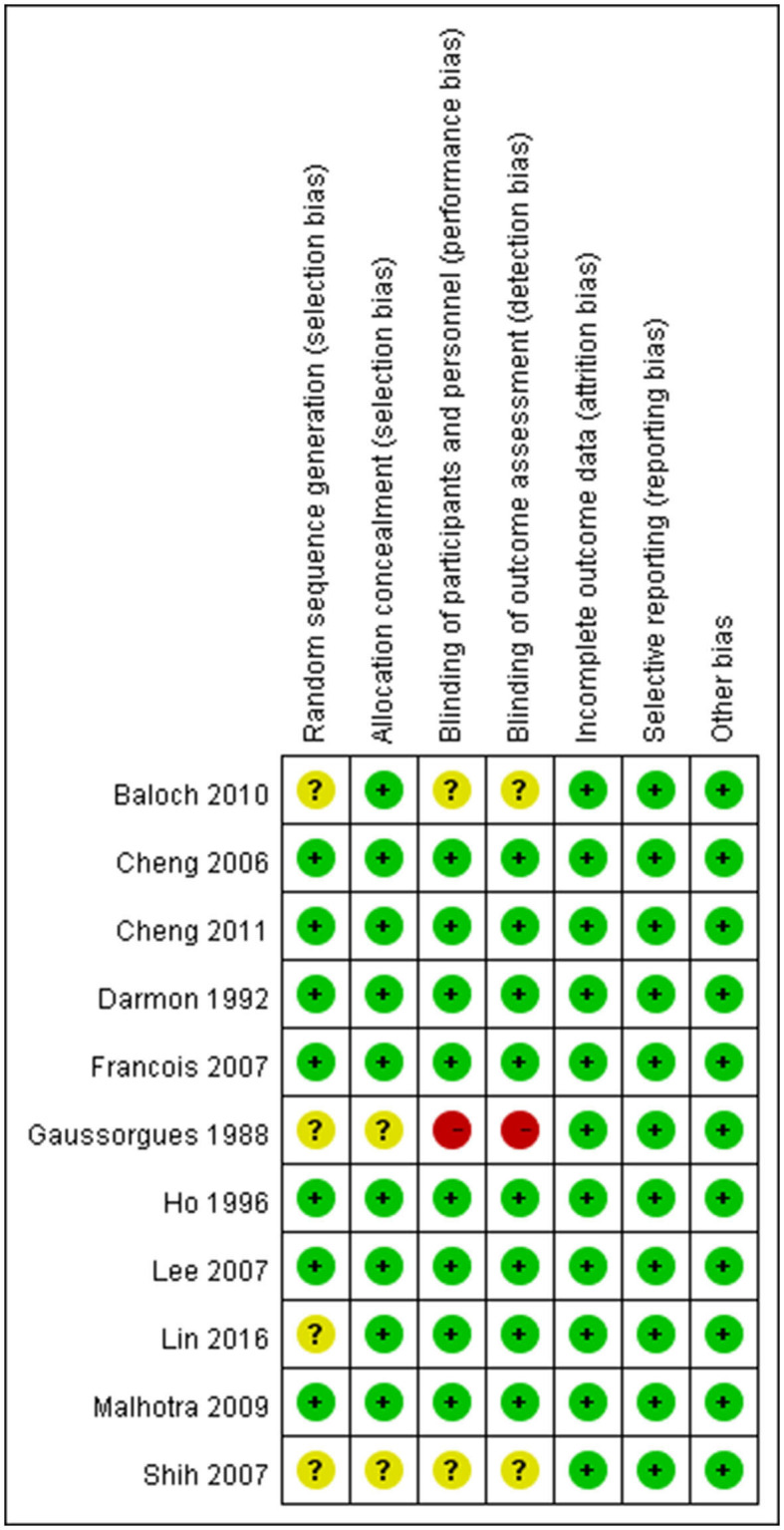
Risk of bias summary: review authors' judgements about each risk of bias item for each included study.

The risk of bias in each domain of each study was low (Figure 3). Control group in Gaussorhues et al.'s study (15) received nothing. This was hard to prevent participants and experimenters to keep unaware the group assignment during study period. High risks were marked in the bias evaluation report. Because no closed-loop was formed for the network of collected postextubation stridor-related literature, inconsistency was not examined here; however, the literature showed a significant publication bias (Egger's test: *p* = 0.013). In contrast, there was no publication bias (Egger's test: *p* = 0.623) in the collected reintubation-related literature.

### Postextubation stridor risk

**Figure 4:**
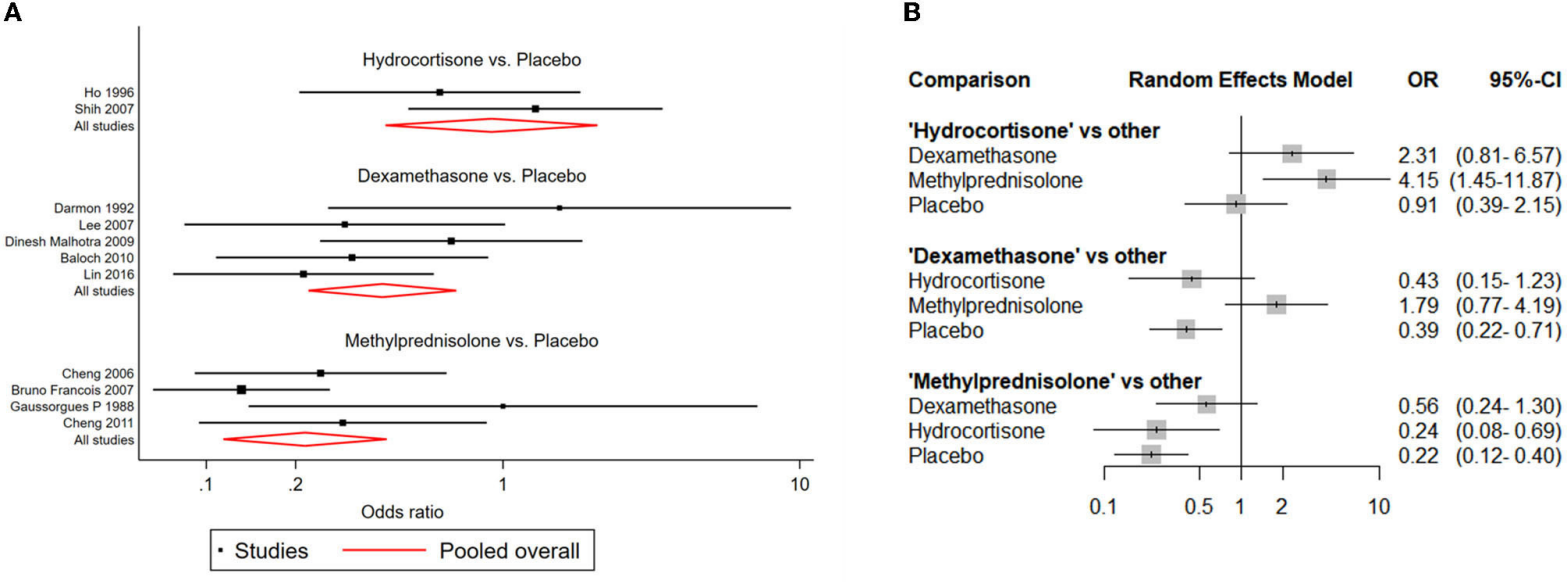
Network forest plots of postextubation stridor. **(A)** Forest plot of individual studies grouped by comparison with direct evidence. ORs and corresponding 95% CIs were displayed. **(B)** Forest plot of summarized association between different corticosteroids and risk of postextubation stridor.

NMAs showed that the risk of postextubation stridor was significantly lower in dexamethasone- and methylprednisolone-treated patients than in placebo-treated patients (dexamethasone: OR: 0.39; 95% CI: 0.22-0.70; methylprednisolone: OR: 0.22; 95% CI: 0.11-0.41) (Table 2A; Figure 4).

**Table 2:** Preventive efficiencies for postextubation stridor (A) and reintubation (B) calculated for each pair of corticosteroids as per the results of the network meta-analyses.

**(A) Postexutbation stridor**			
**Placebo**			
1.09 (0.48,2.49)	Hydrocortisone		
2.55 (1.44,4.53)^*^	2.33 (0.85,6.37)	Dexamethasone	
4.65 (2.47,8.76)^*^	4.25 (1.49,12.10) ^*^	1.82 (0.79,4.19)	Methylprednisolone
**(B) Reintubation**			
**Placebo**			
1.53 (0.52,4.49)	Hydrocortisone		
2.96 (1.18,7.43)^*^	1.93 (0.47,7.97)	Dexamethasone	
2.37 (1.43,3.94)^*^	1.55 (0.47,5.09)	0.80 (0.28,2.29)	Methylprednisolone

Relative effect estimates in pooled ORs and corresponding 95% CIs are displayed for 4 corticosteroids treatments. In the table, each OR represents the interaction intensity of two treatments. Cells with a ^*^indicates a treatment above the cell has a significantly influence than the treatment shown to its right.

### Reintubation risk

**Figure 5:**
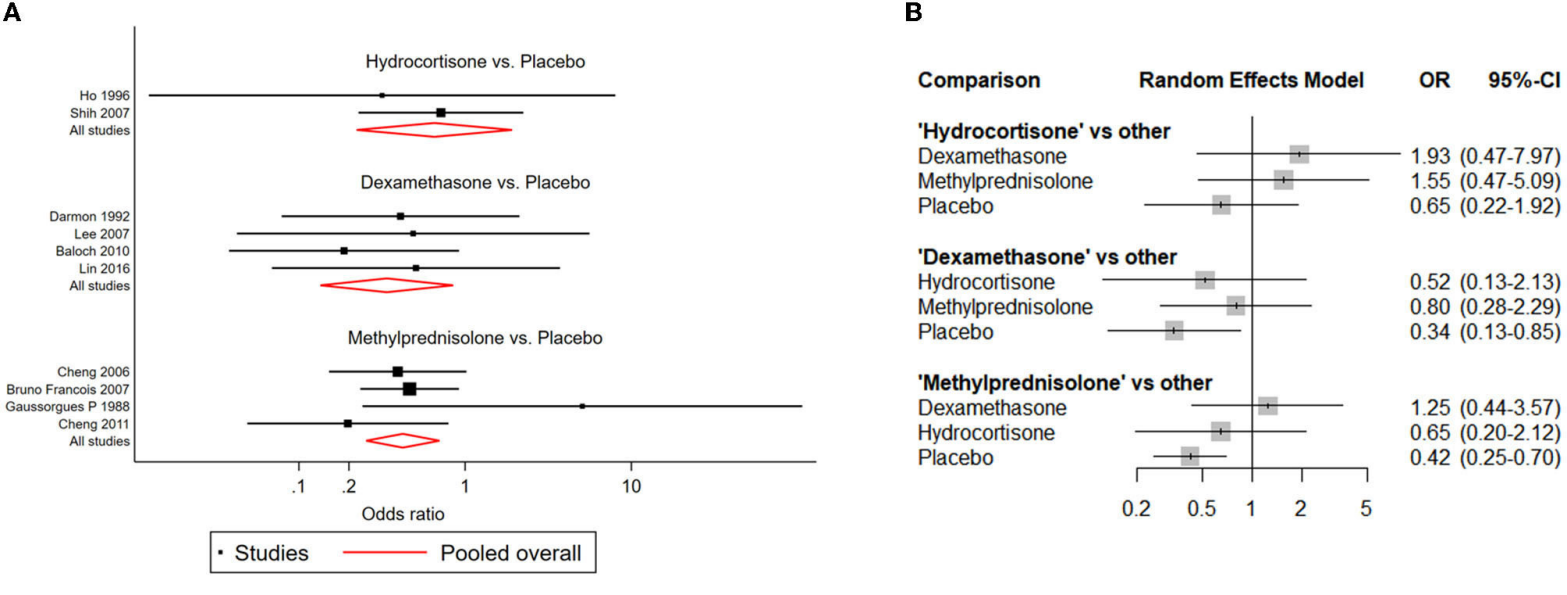
Network forest plots of reintubation. **(A)** Forest plot of individual studies grouped by comparison with direct evidence. ORs and corresponding 95% CIs were displayed. **(B)** Forest plot of summarized association between different corticosteroids and risk of reintubation.

Compared with placebo, both dexamethasone and methylprednisolone were found significantly associated with lower risk of reintubation (dexamethasone: OR: 0.34; 95% CI: 0.13-0.85; methylprednisolone: OR: 0.42; 95% CI: 0.25-0.70) (Table 2B; Figure 5).

### Ranked order of interventions

**Figure 6:**
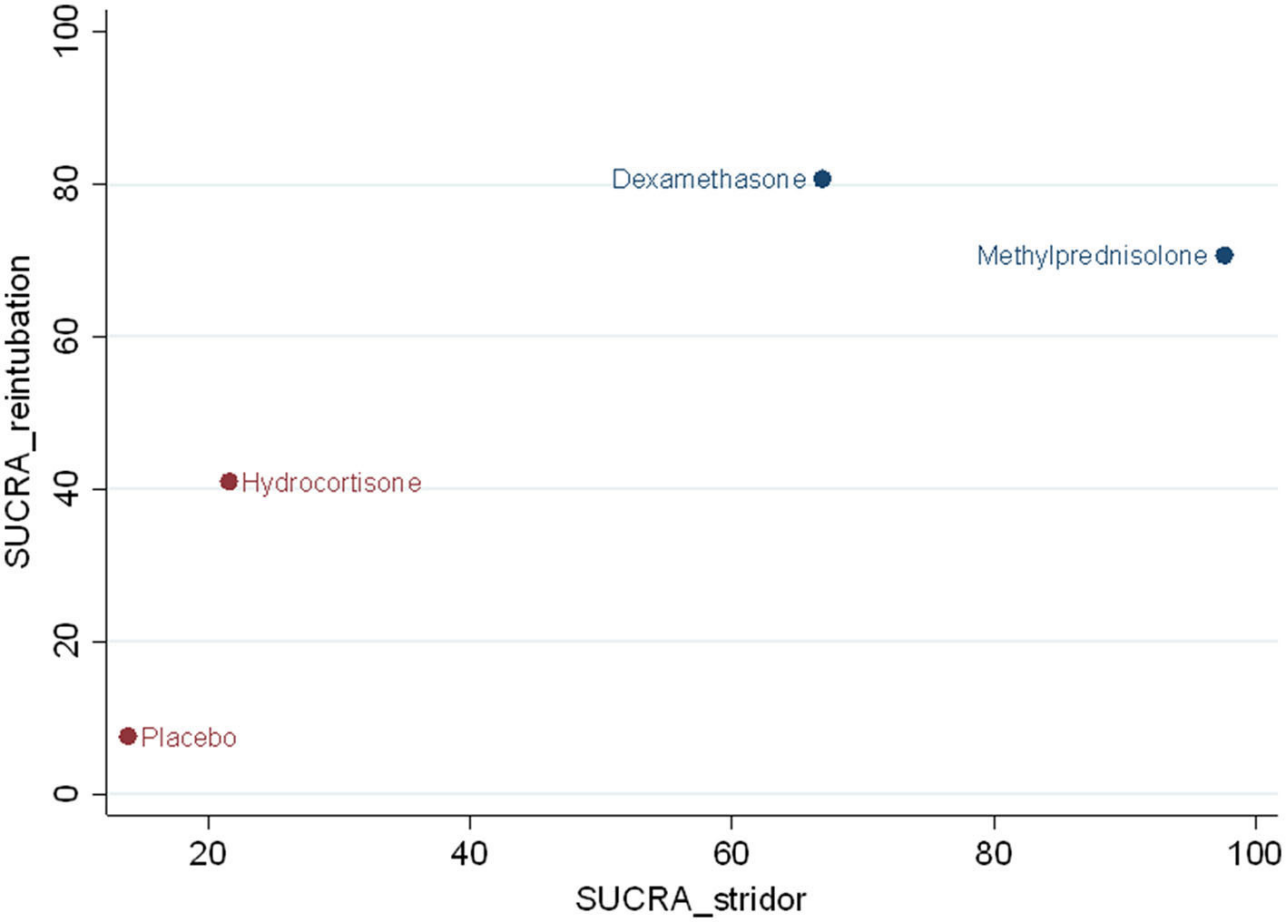
Clustered ranking plot. The SUCRA value for each agent for preventing effect of postextubation stridor and reintubation.

For preventing postextubation stridor, methylprednisolone had the largest surface under the cumulative ranking curve (SUCRA) (97.6), followed by dexamethasone (66.9), hydrocortisone (21.6), and placebo (13.9). For reducing the risk of reintubation, dexamethasone had the largest SUCRA (80.7), followed by methylprednisolone (70.7), hydrocortisone (41.0), and placebo (7.6) (Figure 6). According to the SUCRA ranking outcomes, methylprednisolone and dexamethasone were considered in one with the most potent intervention cluster (Figure 6).

### Subgroup analysis

For patients with positive CLTs, both dexamethasone and methylprednisolone were associated with a significantly lower risk of postextubation stridor than was placebo (dexamethasone: OR:0.26; 95% CI:0.14–0.50; SUCRA: 75.1; methylprednisolone: OR:0.26; 95% CI:0.13–0.55; SUCRA: 74.8). Moreover, both corticosteroids were associated with a significantly lower risk of reintubation than was placebo (dexamethasone: OR:0.31; 95% CI:0.10–0.94; SUCRA: 74.0; methylprednisolone: OR:0.32; 95% CI:0.14–0.69; SUCRA: 74.8) methylprednisolone, respectively.

## Discussion

This is the first network meta-analysis of 11 RCTs with a total of 2,371 patients that compare the efficacies of three commonly used corticosteroids (dexamethasone, methylprednisolone, and hydrocortisone) against postextubation stridor and reintubation in patients with a scheduled extubation. This analysis provided useful information on the choice of appropriate corticosteroid formula for preventing postextubation stridor and reintubation in this population. Of three kinds of corticosteroid, dexamethasone and methylprednisolone were the most efficacious corticosteroids in this network meta-analysis. Methylprednisolone was ranked highest in the preventing postextubation stridor, and in contrast, hydrocortisone was ranked lowest. Regarding reintubation, dexamethasone and methylprednisolone was ranked much higher than hydrocortisone. A similar trend was observed in the subgroup analysis of high risk patients selected using cuff-leak test. Overall, this network meta-analysis also found that methylprednisolone and dexamethasone were associated with significantly lower risk of postextubation stridor and reintubation than placebo among overall population and the subgroups selected with cuff-leak test. In summary, we found that methylprednisolone and dexamethasone are the most potent kind of corticosteroids in the preventing postextubation stridor and reintubation and their prophylactic use can effectively reduce the risk of postextubation stridor and reintubation before elective extubation among patients with mechanical ventilation. In contrast, the usefulness of hydrocortisone was limited in this clinical condition. All these findings should suggest the better role of methylprednisolone and dexamethasone than hydrocortisone in this clinical entity.

Our findings in this network meta-analysis can be explained by the different anti-inflammatory activities of three corticosteroids. Corticosteroids differ in their relative amount of anti-inflammatory potency. Dexamethasone and methylprednisolone have much higher anti-inflammatory potency than hydrocortisone. In general, 25 mg of hydrocortisone is equivalent to 5 mg of methylprednisolone, and 1 mg of dexamethasone according to their relative anti-inflammatory potency (28). Therefore, methylprednisolone and dexamethasone are supposed to more effectively reduce inflammatory laryngeal edema and further significantly lower the risk of postextubation stridor and reintubation than hydrocorticosone. We suggest using methylprednisolone (20 mg ever 6 h) and dexamethasone (5 mg ever 6 h) before extubation.

Our study has some limitations. First, the regimens of the corticosteroid treatments are different in the selected RCTs, which might lead to different preventive effects. Second, the number of enrolled studies is small; thus, we cannot further assess the effects of the different regimens of corticosteroids. Additional studies are needed to investigate their confounding effects. Third, some mechanisms such as publication bias and other forms of reporting bias might have impact on our interpretation of the results. Several possible confounding factors, such as sex, duration of intubation, and the size of the endotracheal tube were not assessed in this meta-analysis. There are some potential biases, heterogeneity or generalizability in meta-analyses. The meta-analysis combines the results of various types of study design to generate an overall effect size. If there are significant heterogeneities in these studies, the focus should shift from the summary effect to the dispersion itself. In addition, meta-analysis focused on the main outcomes and its results can be generalizable to particular group of patients but not another.

However, we could not do a subgroup analysis because so few trials reported separate results based on sex, duration of intubation, and the size of the endotracheal tube. We did not evaluate the risk of adverse effects caused by corticosteroids.

Although the risk of corticosteroid-related complications, e.g., fluid retention, infection, gastrointestinal bleeding, and hyperglycemia are low in additional investigations are needed to assess the possibility of negative side effects. Finally, because studies were not administering equipotent doses of steroids, methylprednisolone and dexamethasone are more effective than hydrocortisone and this may be a significant confounder.

The comparison between different corticosteroids are statistical rather than direct clinical comparisons. However, we clarify the selection of studies, pre-specification of the pooled analysis, the risk of bias in each domain of each study was low and the outcome of individual studies in relation to the pooled result. We believe the validity and interpretation of pooled analyses.

## Conclusion

Our findings indicate that methylprednisolone and dexamethasone are the most effective corticosteroids for preventing postextubation stridor and reintubation. Nevertheless, future large-scale clinical studies are needed to confirm our findings.

## Data availability statement

The original contributions presented in the study are included in the article/Supplementary material, further inquiries can be directed to the corresponding author.

## Author contributions

C-CL, C-MChe, and I-JF: concepts and design. J-WL, K-CC, S-RC, and I-JF: analysis and interpretation of data. C-MCha and C-CL: drafting of the manuscript. C-MChe, K-ML, and Y-TW: critical revision of the manuscript. I-JF: statistical analysis. All authors contributed to the article and approved the submitted version.
