# Supplementary material for: Comparative efficacies of various corticosteroids for preventing postextubation stridor and reintubation: a systematic review and network meta-analysis
Source: Front Med (Lausanne). 2023 Jul 24;10:1135570. doi: 10.3389/fmed.2023.1135570 (PMC10406286; doi:10.3389/fmed.2023.1135570)
Supplement: Supplementary file 1 [file Data_Sheet_1.docx]

**Appendix 1.** Database Search Terms

**Cochrane Library search terms**

#1 Intubation or Intratracheal

#2 Laryngeal edema

#3 Airway obstruction

#4 Stridor*

#5 Post extubation laryn* edema*

#6 Steroid* or corticosteroid* or glucocorticoid*

#7 Prednisone or prednisolone or methylprednisolone or dexamethasone or cortisone or hydrocortisone

#8 (#1 or #2 or #3) and (#4 or #5) and (#6 or #7)

**PubMed search terms**

#1 Intubation or Intratracheal

#2 Laryngeal edema

#3 Airway obstruction

#4 Stridor*

#5 Post extubation laryn* edema*

#6 Steroid* or corticosteroid* or glucocorticoid*

#7 Prednisone or prednisolone or methylprednisolone or dexamethasone or cortisone or hydrocortisone

#8 (#1 or #2 or #3) and (#4 or #5) and (#6 or #7)

**Embase search terms**

#1 Intubation or Intratracheal or Laryngeal edema or Airway obstruction

#2 Stridor* or Post extubation laryn* edema*

#3 Steroid* or corticosteroid* or glucocorticoid* or Prednisone or prednisolone or methylprednisolone or dexamethasone or cortisone or

hydrocortisone

#4 (#1 and #2 and #3) and randomized controlled trials

**Clinicaltrial search terms**

Stridor and interventional studies and adult

Post extubation and interventional studies and adult

**Appendix 2.** The regimen of corticosteroids in each study

| **Study** | **Corticosteroid** | **Route** | **Dose**  **Frequency** | **Dose** | **Treatment periods** | **Frequency** | **Total dose** |
| --- | --- | --- | --- | --- | --- | --- | --- |
| Gaussorgues et al./1988 | methylprednisolone | IV+IM | 2 | 1INJ:40mg+40mg IM | before extubation | One hour before extubation | 80mg |
| Darmon et al./ 1992 | dexamethasone | IV | 1 | 1INJ:8mg | before extubation | One hour before extubation | 8mg |
| Ho et al./ 1996 | hydrocortisone | IV | 1 | 1INJ:100mg | before extubation | One hour before extubation | 100mg |
| Cheng et al./ 2006 | methylprednisolone | IV | 4 | 1INJ:40mg or 4INJ:40mg | before and after extubation | every 6 hrs over 24 hrs | 40mg or 160mg |
| Francois et al./ 2007 | methylprednisolone | IV | 4 | 4INJ:20mg | before and after extubation | 12 h before extubation and every 4 h | 80mg |
| Lee et al./ 2007 | dexamethasone | IV | 4 | 4INJ:5mg | before and after extubation | every 6 hrs on the day preceding extubation | 20mg |
| Shih et al./ 2007 | hydrocortisone | IV | 4 | 4INJ:NR | before and after extubation | every 6 hrs over 24 hrs | NR |
| Malhotra et al./ 2009 | dexamethasone | IV | 4 | 4INJ:8mg | before and after extubation | every 4 hrs prior to planned extubation, at extubation and 6 and 12 hrs after extubation | 32mg |
| Baloch et al./ 2010 | dexamethasone | IV | 4 | 4INJ:5mg | before and after extubation | every 6 hrs over 24 hrs | 20mg |
| Cheng et al./ 2011 | methylprednisolone | IV | 1 | 1INJ:40mg | before extubation | 4 h prior to a planned extubation | 40mg |
| Lin et al./ 2016 | dexamethasone | IV | 4 | 4INJ:5mg or 10mg | before and after extubation | every 6 hrs over 24 hrs | 20mg or 40mg |

IV, intravenous; IM, intramuscular; INJ, injection
